# Factors contributing to the burnout of the faculties of a medical university in Iran: A cross‐sectional study

**DOI:** 10.1002/brb3.3384

**Published:** 2024-01-17

**Authors:** Sulmaz Ghahramani, Mohsen Moghadami, Navid Omidifar, Seyed Mohamad Mehdi Tabatabaei, Mohammad Sayari, Kamran Bagheri Lankarani

**Affiliations:** ^1^ Health Policy Research Center, Institute of Health Shiraz University of Medical Sciences Shiraz Iran; ^2^ Department of Internal Medicine, School of Medicine Shiraz University of Medical Sciences Shiraz Iran; ^3^ Deputy of Education Shiraz University of Medical Sciences Shiraz Iran; ^4^ Department of Mathematical Sciences and Research Methods Centre Durham University Durham UK

**Keywords:** faculty, Iran, professional burnout, psychological well‐being

## Abstract

**Background and aims:**

Faculty members confront a variety of obstacles over time, the most recent of which is the coronavirus disease 2019 pandemic, which may increase their vulnerability to burnout (BO). This study aims to examine BO in medical school faculties, as well as the factors that lead to BO and well‐being in them.

**Methods:**

This cross‐sectional study was conducted in 2021 using online questionnaires completed by 222 faculty members of a medical university in Iran. The Maslach Burnout Inventory‐Human Services Survey (MBI‐HSS) and the Well‐being index (WBI) were used. Additionally, we gathered individual‐level profiles (demographic, well‐being) and occupational information (job profile, attitude toward work).

**Results:**

A total of 60 (27%) faculties reported having high BO, and 112 (50.5%) reported having low well‐being. Being female (odds ratio, OR = 2.69), having time to spend with the family (OR = .26), the intent of turnover (OR = 8.65), job recommendation to the offspring (OR = .26), and experiencing violence last year (OR = 2.97) were some of the individual‐level factors and job‐related attitudes associated with a higher BO. In the neural network for BO, the most important variables were the intention of turnover, followed by adequate family time.

**Conclusion:**

One third of the responding faculty reported severe BO, and BO was found to be significantly associated with lower well‐being. The increased levels of BO and a decreased experience of well‐being were both associated with a higher intention of turnover. According to the study, it is important to pay attention to both clinical and nonclinical field faculty members, female faculty members, those who have a high workload, and members who have experienced violence in the workplace. By acknowledging the unique challenges and experiences faced by these individuals, tailored measures can be developed to address their specific concerns and foster a supportive and inclusive environment.

## Graphical abstract

This cross‐sectional study was conducted in 2021 using online questionnaires completed by 222 faculty members of a medical university in Iran. One third of the responding faculties reported severe BO, and BO was found to be significantly associated with low well‐being. By acknowledging the unique challenges and experiences faced by these individuals, tailored measures can be developed to address their specific concerns and foster a supportive and inclusive environment.

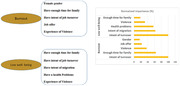

## 1 BACKGROUND

Medical faculties play a key role in increasing the quality of higher medical education and the training of expert human resources for the health system. Competent and eager medical faculties can aid learners in progressing physically, mentally, emotionally, socially, and spiritually (Karimi et al., 2013).

All institutions, including academic medicine ones, should rely on energetic, motivated, and committed professors for their success (Abdo et al., 2015). Moreover, faculty well‐being is vital for the success of these institutions. Medical faculties are involved in education, research, personal development, and, in many instances, providing health care. Along with the resource constraint, medical faculties struggle with flux issues over time. First, there is the quickly changing scientific literature, which demands significant efforts to stay informed, and second, there is the process and documentation needed for academic promotion and self‐development, which also require a great effort and a high workload, as well as a challenging work environment. A recent addition to these challenges is the coronavirus disease 2019 (COVID‐19) pandemic. This pandemic transformed the techniques of education toward virtual learning, and coping with this circumstance is even more challenging for faculties (Bdair, 2021; Nimavat et al., 2021). Furthermore, the clinical field faculties faced higher workloads and mental pressure during the pandemic as they were concerned with providing care to the patients (Ghahramani et al., 2021), whereas they were also worried about their health, the health of their learners, and the health of their loved ones. All these demanding circumstances lead to a rising rate of stress, poor well‐being, and burnout (BO) among medical faculty members. BO is a three‐dimensional affective response to chronic work‐related stress that is not effectively handled (Chirico, Nucera, et al., 2022). It comprises emotional exhaustion (EE), depersonalization (DP), and a loss of personal accomplishment (PA) (Schutte et al., 2000). EE arises when employees feel fatigued or have insufficient energy to participate emotionally. DP comprises developing negative attitudes and thoughts toward people who perform tasks for them. Those who experience reduced PA tend to underestimate their skills to carry out tasks and interact with others. This scenario is a result of both individual traits and work‐related factors, most notably workload (Ghahramani et al., 2022; Khamisa et al., 2013; Kisely et al., 2020; Lim et al., 2010; Liu et al., 2020; Manzano García & Ayala Calvo, 2021; Montgomery et al., 2015). Not only workload but also other organizational factors, workplace violence, and emotional demands may cause BO syndrome (Chirico, Afolabi, et al., 2022). BO has a detrimental effect on not only the health and social relationships of the affected individual but also on the quality of the services offered (Poghosyan et al., 2010; Shanafelt & Noseworthy, 2017; Williams et al., 2007). BO is more prevalent in jobs where individuals spend more time assisting others, such as healthcare workers, psychologists, social workers, teachers, and other helping professionals (Chirico, Afolabi, et al., 2021; Chirico, Capitanelli, et al., 2021; Chirico, Crescenzo, et al., 2021; Ogungbamila, 2013).

One third of all medical faculties have been reported to have BO, with the prevalence even greater in clinical field faculties (Arvandi et al., 2016; Dandar et al., 2019). Although it is well established that both individual‐level and work‐related factors influence an employee's BO (Arvandi et al., 2016; Ghahramani et al., 2022; Stoyanov, 2014), previous BO studies among faculties have primarily focused on the prevalence and predictors of BO (Dandar et al., 2019; Haghighinejad et al., 2021; Mansourian et al., 2019), and analyses of predictors of BO across all medical faculties did not consider the effect individual‐level profiles (demographic, subjective well‐being) and occupational situation (job profile, attitude toward work) in most instances. There are few if any, studies that have evaluated the BO of faculty members during the COVID‐19 epidemic through a structured approach. Understanding the situation with BO and the characteristics of high‐risk groups will provide important evidence for health officials to improve screening methods for vulnerable faculty members and to apply proactive holistic measures without delay (Heath et al., 2020; Trumello et al., 2020). Thus, the purpose of this study was to examine the prevalence of BO in all faculties (academic members) employed at Shiraz University of Medical Sciences, as well as the predictors of BO and well‐being in these faculties.

## 2 METHODS

### 2.1 Study setting and population

This cross‐sectional study included all faculties (academic members) employed at Shiraz University of Medical Sciences in Iran during the study period. The study was undertaken in the year 2021, from February to September, during a period of steady COVID‐19 pandemic status.

### 2.2 Study procedure

This study consisted of two distinct stages. In the initial phase, a trustworthy staff with a prestigious background respected by the faculties was identified in each department to notify research participants about the investigation. This staff dubbed the “Trusted Employee,” briefs faculty members about the study in an easy‐to‐understand manner. These approaches include writing letters, making phone calls, meeting in person, and browsing university websites. It was aimed at helping faculties in all departments and units understand the study process. These include those engaged in research, basic science, and clinical care. The path to participation in the survey was documented by a project's trusted employee and then informed to the research group.

In the second step, a questionnaire was issued to faculties via a secure online platform with a link to respond. We stated on the questionnaire that the current study is a collaborative effort to ascertain faculty members’ well‐being and BO. As participants must not have unreasonable expectations during this step, it was emphasized that this is an anonymous survey with no relation to individuals’ administrative decisions. We used the census sampling method, so questionnaires were delivered to all enrolled faculty members, and a two‐time reminder was sent via short message. The trusted employee maintained a record of the progress of the questionnaires delivered and collected.

Additionally, the participants were assured that the information was fully confidential and that no personal information was shared with third parties. To maintain the confidentiality of the participants, questionnaires were gathered anonymously.

### 2.3 Instrument of investigation

We gathered individual‐level profiles (demographic, subjective well‐being) and occupational information (job profile, attitude toward work). Two valid and reliable questionnaires were used in the study: the Maslach Burnout Inventory‐Human Services Survey (MBI‐HSS) in Persian and the Well‐being iindex (WBI).

The MBI‐HSS in Persian (Moalemi et al., 2018) as the gold standard for determining the presence of BO was used. This 22‐item questionnaire assessed 3 dimensions: EE, DP, and PA, and was evaluated on a scale ranging from never (0) to daily (6) (viz., 7 points for each item). Thus, faculty members might convey their emotions using the given options. Thus, higher EE and DP scores and lower PA ratings may indicate a higher level of BO. EE was classified as low, medium, or high based on scores of less than or equal to 16, 17–27, and 27 and higher. For DP, 3 groups were identified based on their scores: 6 and less than 6, 7–12, and 13 and above. Finally, the PA was classified as low, medium, or high using the following scores: 31 and below, 38–32, and 39 and above. For modeling purposes, a score greater than or equal to 27 on the EE or greater than or equal to 13 on the DP might be regarded as having at least one symptom of BO (Moalemi et al., 2018). Cronbach's alpha for MBI‐HSS in this study population was .70, and for EE, DP, and PA dimensions, it was .88, .70, and .81, respectively.

The WBI was first developed to assess physician distress (Shanafelt et al., 2014); however, it has been demonstrated that this tool may be used to examine other health system employees as well (Shanafelt & Noseworthy, 2017). This tool comprises seven yes‐or‐no questions. No received a score of 0, whereas Yes received a score of 1. The scoring threshold is 4, which implies that those with a score of 4 and above are considered to be in low well‐being, whereas those with a score of less than 4 are considered to be in good well‐being. This index demonstrated acceptable reliability in our investigation, with an overall Cronbach's alpha of .8.

In the occupational information section, faculty members were classified into two categories in the employment profile part of the questionnaire: those engaging in patient care and those practicing in basic science or research faculties referred to as nonclinical. Additionally, a positive history of suicide and being a victim of violence (verbal, physical, and emotional) in the workplace during the preceding year was investigated.

Faculty members were asked, “have you considered changing jobs in the last three months?” “have you considered changing wards in the last three months?,” and “have you considered migrating to another country in the last three months?” These questions may reveal an employee's attitude toward his or her current job.

### 2.4 Statistical analysis

In this research, a two‐stage hybrid procedure was applied to evaluate the effects of variables related to BO and low well‐being. In the first stage, robust logistic regression was used to identify the significant variables. In the second stage, the significant variables were entered into the neural network (NN) model to extract their importance (Lin, 2009). The NN can detect both linear and nonlinear relationships between exploratory and response variables, a capability that is not available in logistic regression analysis. On the contrary, due to the “black box” nature of the NN, it fails to test the relationships. Therefore, the combination of these two methods produces a procedure capable of testing relationships and capturing both linear and nonlinear relationships. Multivariate logistic regression analysis was carried out using STATA (version 17). The NN procedure with multilayer perceptrons was performed using SPSS 26. The correlation between BO and well‐being was assessed using Pearson's correlation. The significance level was set at 0.05.

#### 2.4.1 Ethics approval and consent to participate

The ethics committee of Shiraz University of Medical Sciences approved this study, and the ethical code is IR.SUMS.REC.1399.1106. All procedures performed in this study followed the ethical standards of the institutional research committee and with the 1964 Helsinki Declaration and its later amendments or comparable ethical standards. Informed consent was obtained from all individual participants included in the study.

## 3 RESULTS

### 3.1 The dataset

Out of the 994 faculty members, 222 responded completely to the questionnaire. This sample size seems adequate when calculating the optimum sample size of the study, with the margin of error being between 5% and 6% (http://www.raosoft.com/samplesize.html). The subgroups of qualitative variables and their frequencies among 222 faculty members are represented in Table 1. As can be seen, 99 faculty members (44.6%) were male, and 188 members (84.7%) were married. Additionally, 116 (52.3%) participants had moderate‐to‐severe EE experiences, 60 (27%) people had severe BO, and 112 (50.5%) members had low‐stated well‐being. Table 2 also includes the most frequently used descriptive statistics for quantitative variables. The mean of BO dimensions and well‐being is shown in Table 3 by gender, department, work status, and on‐call status. Additionally, Table 4 presents gender, department, job, and on‐call frequency in relation to the severity of BO aspects and well‐being. A total of 27 (23.8%) clinical field faculty members reported having a high BO, 57 (50.4%) reported having a moderate‐to‐high EE, and 66 (58.4%) reported having a low experience of well‐being.

**TABLE 1:** The frequency of qualitative variables (*N* = 222).

Variable	Subgroups	Frequency	Percent
Burnout (dependent variable)	Do not have	162	73.0
	Have	60	27.0
Low well‐being (dependent variable)	Do not have	110	49.5
	Have	112	50.5
Gender	Male	123	55.4
	Female	99	44.6
Marital status	Unmarried	28	12.6
	Married	188	84.7
	Divorced or widow	6	2.7
Employment	Official	79	35.6
	Conventional	79	35.6
	Contractual	16	7.2
	Compulsory service	48	21.6
Full time job	Full time faculty	201	90.5
	Non‐full time faculty	21	9.5
Department	Clinical field	113	50.9
	Nonclinical field	109	49.1
Enough time for family^a^	Never or rarely	141	63.5
	Quite often or very often	81	36.5
Number of death patients^b^	Three or less	86	76.1
	Between 4 and 10	11	9.7
	11 or more	16	14.2
Intent of turnover	Yes	81	36.5
	No	141	63.5
Ward change	Yes	70	31.5
	No	152	68.5
Intent of migration	Yes	114	51.4
	No	108	48.6
Job offer^c^	Yes	107	48.2
	No	115	51.8
Health problems	Yes	74	33.3
	No	148	66.7
Suicide	Yes	11	5.0
	No	211	95.0
Violence	Yes	79	35.6
	No	143	64.4
Managerial responsibility	Have	91	41.0
	Not have	127	57.2
Have other monetary resources	Yes	27	12.2
	No	193	86.9
The severity of EE	Low EE	106	47.7
	Moderate EE	56	25.2
	High EE	60	27.0
The severity of DP	Low DP	196	88.3
	Moderate DP	21	9.5
	High DP	5	2.3
The severity of PA	Low PA	65	29.3
	Moderate PA	42	18.9
	High PA	115	51.8
Job satisfaction after COVID‐19 pandemic	No difference	111	50.0
	Become less	95	42.8
	Become more	16	7.2

Abbreviations: COVID‐19, coronavirus disease 2019; DP, depersonalization; EE, emotional exhaustion; PA, personal accomplishment.

^a^ Have you enough free time to spend with your own family.

^b^ In clinical field faculties, do you see patients who die during your working days?

^c^ Would you recommend your children pursue your career?

**TABLE 2:** The common descriptive statistics for quantitative variables (*N* = 222).

Variable	Minimum	Maximum	Mean	Std. deviation
EE	1	54	20.00	12.37
DP	0	22	2.35	3.56
PA	8	48	36.37	9.01
Well‐being scores	0	7	3.46	2.25
Age	30	66	44.51	7.94
Number of on‐call (monthly)	0	30	3.06	5.89
Faculty years of experience	0	35	10.67	8.69

Abbreviations: DP, depersonalization; EE, emotional exhaustion; PA, personal accomplishment.

**TABLE 3:** The mean of burnout (BO) dimensions and well‐being based on gender, department, employment, and on‐call.

		EE	DP	PA	Well‐being
Gender	Male	19.15	2.74	35.72	3.24
	Female	21.07	1.87	37.18	3.74
Department	Clinical	21.68	2.65	35.61	3.81
	Nonclinical^a^	18.27	2.04	37.16	3.10
Employment	Official	21.16	2.46	35.48	3.09
	Conventional	20.00	2.57	36.39	4.06
	Contractual	18.19	1.44	39.25	2.69
	Compulsory service	18.71	2.13	36.83	3.35
On‐call	Have on‐call	18.23	2.38	38.56	3.56
	No on‐call	21.01	2.34	35.13	3.41
Full time job	Full time	19.74	2.34	36.51	3.39
	Full time non‐geographic	22.52	2.48	35.00	4.19

Abbreviations: DP, depersonalization; EE, emotional exhaustion; PA, personal accomplishment.

^a^ Nonclinical field faculties include basic science or research faculties.

**TABLE 4:** The frequencies of gender, department, employment, and on‐call based on severity of burnout (BO) dimensions and well‐being.

		EE			DP			PA			Well‐being	
		Low	Moderate	High	Low	Moderate	High	Low	Moderate	High	Normal	Low
Gender	Male	59	29	35	106	12	5	33	22	68	65	58
	Female	47	27	25	90	9		32	20	47	45	54
Department	Clinical	56	30	27	99	10	4	34	21	58	47	66
	Nonclinical^a^	50	26	33	97	11	1	31	21	57	63	46
Employment	Official	43	21	15	71	6	2	23	15	41	46	33
	Conventional	35	22	22	69	8	2	21	16	42	29	50
	Contractual	8	5	3	14	2		3	5	8	10	6
	Compulsory service	20	8	20	42	5	1	18	6	24	25	23
On‐call	Have on‐call	28	27	25	68	10	2	24	13	43	42	38
	No on‐call	78	29	35	128	11	3	41	29	72	68	74
Full time job	Full time	92	52	57	175	21	5	61	39	101	101	100
	Full time non‐geographic	14	4	3	21	0	0	4	3	14	9	12

Abbreviations: DP, depersonalization; EE, emotional exhaustion; PA, personal accomplishment.

^a^ Nonclinical field includes basic science or research faculties.

In nonclinical field faculties, high BO was observed in 33 (30.3%), moderate‐to‐high EE was observed in 59 (54.1%), and low well‐being was observed in 46 (42%). The Pearson correlation between BO and well‐being score was statistically significant (*r* .72, *p* < .001).

### 3.2 Multivariate logistic regression

The results of significant variables based on the odds ratio (OR) for high BO and low well‐being are illustrated in Tables 5 and 6, respectively. For BO, female faculty compared to male faculty had a higher likelihood of BO (OR = 2.69). Faculty members who spent more time with their families had a lower likelihood of BO (OR = .26). Faculty members were more likely to have BO when they had the intent of turnover (OR = 8.65). They also had a lower likelihood of BO when they recommended their job to the offspring (OR = .26). Faculty members who experienced violence compared to those who did not have significantly higher odds of BO (OR = 2.97).

**TABLE 5:** The results of robust multivariate logistic regression for burnout.

variables (reference)	Odds ratio	Std. error	*p*‐Value	95% [confidence interval]	
**Gender** (male)					
Female	2.69	1.37	.048	1.007	7.196
**Enough time for family** (never or rarely)					
Quite often or very often	.264	0.134	.009	.098	.712
**Intent of turnover** (no)					
Yes	8.654	4.354	.000	3.228	23.198
**Job offer** (no)					
Yes	.263	0.129	.007	.101	.688
**Violence** (no)					
Yes	2.968	1.340	.016	1.225	7.189

**TABLE 6:** The results of robust multivariate logistic regression for low well‐being.

Variables (reference)	Odds ratio	Std. error	*p*‐Value	95% [confidence interval]	
**Enough time for family** (never or rarely)					
Quite often or very often	.447	0.165	.029	.217	.922
**Intent of turnover** (no)					
Yes	6.229	2.785	.000	2.594	14.961
**Intent of migration** (no)					
Yes	2.280	0.893	.035	1.058	4.912
**Health problems** (no)					
Yes	3.117	1.341	.008	1.341	7.244
**Violence** (no)					
Yes	2.629	1.047	.015	1.205	5.737

For low well‐being, faculty members who spent more time with their families were less likely to have low well‐being (OR = .44). Faculty members were more likely to have low well‐being when they had the turnover intention (OR = 6.23). Moreover, a higher likelihood of low well‐being was seen in faculties with the intention of migration (OR = 2.28). Faculty members with known health problems had higher odds of low well‐being (OR = 3.12). Faculty members who experienced violence, compared to others, were more likely to have low well‐being (OR = 3.26).

The complete results of the multivariate logistic regression analysis are provided in Tables S1 and S2.

### 3.3 Neural network analysis

The variables in Tables 5 and 6 were entered into the model as inputs. Furthermore, 10‐fold cross‐validation was performed to evaluate the accuracy of the models. The values of the root mean squared error (RMSE) were estimated for training and testing datasets for 10 networks of each model. The values of RMSE are provided in Table S3. The consistent values of RMSE for training and testing indicate reliable fits for the two models. The importance of input variables in terms of normalized importance is shown in Table 7. For BO, the intent of turnover is the most important variable, followed by enough time for family. For low well‐being, the intent of turnover is the key variable, followed by the intent of migration.

**TABLE 7:** Neural network analysis: The importance of variables.

Dependent variable	Independent variable	Importance	Normalized importance (%)
Burnout	Intent of turnover	0.416	100.00
	Enough time for family	0.265	63.60
	Violence	0.154	37.00
	Job offer	0.095	22.90
	Gender	0.069	16.70
Low well‐being	Intent of turnover	0.371	100.00
	Intent of migration	0.232	62.50
	Health problems	0.211	56.90
	Violence	0.118	31.80
	Enough time for family	0.068	18.30

## 4 DISCUSSION

The purpose of this study was to determine the prevalence of BO among faculty members as well as the most significant predictors of BO and well‐being. The study's findings suggested that one third of responding faculties had severe BO and half had moderate‐to‐severe EE; also, more than half of the participating faculties were not in a state of good well‐being. This prevalence is greater than what was obtained in a study that utilized a single‐item question for BO and found that the prevalence of at least one BO symptom was 29% among US faculty members between 2016 and 2018 (Dandar et al., 2019). Even though in 2015, severe EE was determined to be 6% (Mansourian et al., 2021) and 25% (Arvandi et al., 2016) prevalent in dental, internal medicine, and surgical faculties, respectively, and we do not have a baseline for all faculties before COVID‐19 to compare BO prevalence with currently in Iran. Faculty members’ general well‐being and the prevalence of BO have risen to the point that they are now comparable to the prevalence of BO in healthcare providers who are exposed to high levels of stress and are responsible for patient care in hospitals (Ghahramani et al., 2022).

For the decrease of BO among healthcare workers (HCWs), some scholars have recently focused on interventions for spiritual well‐being. A systematic review found that spiritual‐based health promotion programs in the workplace prevent and/or reduce BO syndrome in HCWs; however, there is a need for more convincing evidence (Chirico, Batra, et al., 2023). The underlying rationale is that organizational values and culture can be represented by workplace spirituality as a framework for promoting employee well‐being. Integrating spirituality into workplace health promotion (WHP) programs can help employees receive support from their organizations to manage BO, work‐related stress, violence, and other psychosocial occupational risk factors (Chirico, Acquadro Maran, et al., 2023).

The “Total Worker Health” strategy will become ever more necessary in the workplace, especially in light of the COVID‐19 pandemic. A strategy that combines WHP programs with workplace safety and health hazard mitigation enhances employee well‐being (Chirico, Sacco, et al., 2021). WHP programs, which help workers and employers while cutting costs, have recently been stated as essential for advancing inclusive and holistic public health. This program may involve lifestyle changes, psychological well‐being, and disability management (Di Prinzio et al., 2022).

BO and low well‐being were found to be prevalent in both clinical and nonclinical field faculty members in this study. However, the prevalence of BO was slightly greater in nonclinical field faculties, but the prevalence of low well‐being was more prevalent in clinical field faculties. Faculty subgroups have previously been shown to differ in their prevalence of BO, with 26% of basic science faculty and 31% of clinical field faculty reporting the condition (Dandar et al., 2019). There was a lower prevalence of severe EE among Iranian nonclinical field faculty members compared to the results of the current investigation (Kaveh et al., 2020), but the prevalence in Iranian clinical field faculty members was equivalent to that identified in our study (Arvandi et al., 2016); moreover, in Iranian nonclinical field faculty members, lower prevalence compared to results of the current study was.

In a prior study, the clinical field faculty in our environment had more exposure to BO than the basic scientific faculty (Haghighinejad et al., 2021). For that study, clinical field faculty members outnumbered fundamental science faculty members by a factor of four, but this was not the case in our investigation (113 and 109 in clinical vs. nonclinical field faculties, respectively). To a large extent, differences in the outcomes of studies comparing clinical and nonclinical field faculties may be due to the use of various study techniques and varied BO cut‐offs in these studies. The COVID‐19 epidemic has brought additional obstacles for the medical education system, students, and faculty, although all of these investigations were conducted before the pandemic (Karimian et al., 2021; Papapanou et al., 2021).

This study revealed that the BO has several correlated factors, including both individual‐level characteristics and job‐related attitudes. Both psychological and individual characters and work‐related profiles have been shown to influence the BO in other studies (Dandar et al., 2019; Ghahramani et al., 2022; Mansourian et al., 2019; Rothenberger, 2017; Stoyanov, 2014).

In this study, having time to spend with the family, the WBI, and the intent of turnover were factors highly associated with a higher BO. Having time to spend with the family could indicate workload (job demand), which has been shown to have a substantial correlation with all BO aspects in the workplace (Alarcon, 2011). In other words, a high workload, which warrants the reduction and justification of job demand, leads to both health problems and BO (Schaufeli & Bakker, 2004). Both low well‐being and the intention of turnover are more likely outcomes of high BO in the workplace than their precursors (Alarcon, 2011; Schaufeli & Bakker, 2004).

A higher likelihood of BO in faculty physicians who have intentions to quit their current position has been found before (Shanafelt et al., 2009). The intention of turnover, which shows attitudes about the work environment, was associated with all dimensions of BO (Alarcon, 2011).

In the wake of the COVID‐19 pandemic, this is one of the first studies to look at the BO and well‐being of medical faculty members. A cross‐sectional study like this one is limited in its ability to conclude about what causes a person's BO and well‐being. Due to the low response rate of faculty members, the findings should be treated with caution, as non‐responding faculty members may have different characteristics than respondents. Additional multicenter studies with a larger sample size on contributing factors are necessary.

## 5 CONCLUSION

One third of the responding faculty reported severe BO, and BO was found to be significantly associated with lower well‐being. The increased levels of BO and a decreased experience of well‐being were both associated with a higher intention of turnover. According to the study, it is important to pay attention to both clinical and nonclinical field faculty members, female faculty members, those who have a high workload, and members who have experienced violence in the workplace. An implication for health policymakers could be the “Total Worker Health” strategy that combines WHP programs with workplace safety and health hazard mitigation to enhance employee well‐being.

## AUTHOR CONTRIBUTIONS

**Sulmaz Ghahramani**: Investigation; methodology; formal analysis; writing—original draft preparation; writing—review and editing. **Mohsen Moghadami**: Investigation; project administration; methodology; supervision; writing—original draft preparation; writing—review and editing. **Navid Omidifar**: Investigation; project administration; methodology; supervision; writing—original draft preparation; writing—review and editing. **Seyed Mohamad Mehdi Tabatabaei**: Investigation; supervision; methodology; writing—original draft preparation; writing—review and editing. **Mohammad Sayari**: Formal analysis; writing—original draft preparation; writing—review and editing. **Kamran Bagheri Lankarani**: Conceptualization; supervision; writing—original draft preparation; writing—review and editing.

## CONFLICT OF INTEREST STATEMENT

The authors have no relevant conflicts of interest to disclose.

## FUNDING INFORMATION

No funding was received for conducting this study.

### PEER REVIEW

The peer review history for this article is available at https://publons.com/publon/10.1002/brb3.3384.

## Supporting information

Appendixes
Table S1 The results of robust multivariate logistic regression for burnout.
Table S2 The results of robust multivariate logistic regression for low well‐being.
Table S3 RMSE for the neural network.

## Data Availability

The datasets generated during and/or analyzed during the current study are available from the corresponding author upon reasonable request.
