# Supplementary material for: Factors contributing to the burnout of the faculties of a medical university in Iran: A cross‐sectional study
Source: Brain Behav. 2024 Jan 17;14(1):e3384. doi: 10.1002/brb3.3384 (PMC10794124; doi:10.1002/brb3.3384)
Supplement: Supplementary file 1 — Appendixes Table S1 The results of robust multivariate logistic regression for burnout. Table S2 The results of robust multivariate logistic regression for low well‐being. Table S3 RMSE for the neural network. [file BRB3-14-e3384-s001.docx]

**Supplementary appendix**

**Supplement to:** Factors contributing to the burnout and wellbeing of faculties

**Table S1. The results of robust multivariate logistic regression for burnout.**

| variables (Reference) | Odds ratio | Std. error | z | P-value | 95% Confidence Interval] | |
| --- | --- | --- | --- | --- | --- | --- |
| Age | 0.93 | 0.04 | -1.630 | 0.102 | 0.849 | 1.015 |
| number of on-call | 1.06 | 0.05 | 1.260 | 0.207 | 0.967 | 1.167 |
|  |  |  |  |  |  |  |
| Gender (male) |  |  |  |  |  |  |
| female | 2.69 | 1.37 | 1.970 | 0.048 | 1.007 | 7.196 |
|  |  |  |  |  |  |  |
| Marital (unmarried) |  |  |  |  |  |  |
| married | 2.39 | 1.84 | 1.110 | 0.267 | 0.526 | 10.187 |
| divorced or widow | 3.50 | 5.73 | 0.750 | 0.456 | 0.146 | 73.419 |
|  |  |  |  |  |  |  |
| Employment (official) | |  |  |  |  |  |
| Conventional | 1.47 | 0.80 | 0.770 | 0.442 | 0.529 | 4.295 |
| Contractual | 0.13 | 0.15 | -1.770 | 0.077 | 0.015 | 1.238 |
| Compulsory service | 0.37 | 0.30 | -1.200 | 0.229 | 0.077 | 1.850 |
|  |  |  |  |  |  |  |
| full time job (full time) | |  |  |  |  |  |
| full time non-geographic | 0.77 | 0.63 | -0.290 | 0.775 | 0.163 | 3.871 |
|  |  |  |  |  |  |  |
| Department (clinical) | |  |  |  |  |  |
| Non-clinical | 1.41 | 0.74 | 0.640 | 0.523 | 0.502 | 3.876 |
|  |  |  |  |  |  |  |
| Enough time for family (never or rarely) | | |  |  |  |  |
| quite often or very often | 0.264 | 0.134 | -2.630 | 0.009 | 0.098 | 0.712 |
|  |  |  |  |  |  |  |
| Intent of turnover (no) | |  |  |  |  |  |
| yes | 8.654 | 4.354 | 4.290 | 0.000 | 3.228 | 23.198 |
|  |  |  |  |  |  |  |
| Ward change (no) | |  |  |  |  |  |
| yes | 1.722 | 0.830 | 1.130 | 0.259 | 0.670 | 4.427 |
|  |  |  |  |  |  |  |
| Intent of migration (no) | |  |  |  |  |  |
| yes | 1.426 | 0.718 | 0.710 | 0.481 | 0.532 | 3.826 |
|  |  |  |  |  |  |  |
| Job offer to offspring (no) |  |  |  |  |  |  |
| yes | 0.263 | 0.129 | -2.720 | 0.007 | 0.101 | 0.688 |
|  |  |  |  |  |  |  |
| Health problems (no) | |  |  |  |  |  |
| yes | 2.653 | 1.321 | 1.960 | 0.050 | 1.000 | 7.039 |
|  |  |  |  |  |  |  |
| Suicide (no) |  |  |  |  |  |  |
| yes | 0.380 | 0.427 | -0.860 | 0.390 | 0.042 | 3.442 |
|  |  |  |  |  |  |  |
| Violence (no) | |  |  |  |  |  |
| yes | 2.968 | 1.340 | 2.410 | 0.016 | 1.225 | 7.189 |
|  |  |  |  |  |  |  |
| Number of death patients (3 or less) | |  |  |  |  |  |
| between 4 to 10 | 0.104 | 0.134 | -1.760 | 0.078 | 0.008 | 1.287 |
| 11 or more | 1.869 | 1.791 | 0.650 | 0.514 | 0.286 | 12.228 |
|  |  |  |  |  |  |  |
| constant | 0.636 | 1.548 | -0.190 | 0.852 | 0.005 | 75.183 |

**Table S2. The results of robust multivariate logistic regression for low well-being.**

| variables (Reference) | Odds ratio | Std. error | z | P-value | 95% Confidence Interval] | |
| --- | --- | --- | --- | --- | --- | --- |
| Age | 0.965 | 0.030 | -1.140 | 0.255 | 0.907 | 1.026 |
| number of on-call | 0.978 | 0.040 | -0.550 | 0.585 | 0.902 | 1.060 |
|  |  |  |  |  |  |  |
| Gender (male) |  |  |  |  |  |  |
| female | 2.199 | 0.887 | 1.950 | 0.051 | 0.998 | 4.846 |
|  |  |  |  |  |  |  |
| Marital (unmarried) |  |  |  |  |  |  |
| married | 2.973 | 1.833 | 1.770 | 0.077 | 0.888 | 9.952 |
| divorced or widow | 4.322 | 5.930 | 1.070 | 0.286 | 0.294 | 63.611 |
|  |  |  |  |  |  |  |
| Employment (official) | |  |  |  |  |  |
| Conventional | 1.462 | 0.665 | 0.830 | 0.404 | 0.599 | 3.566 |
| Contractual | 0.504 | 0.431 | -0.800 | 0.423 | 0.094 | 2.692 |
| Compulsory service | 0.792 | 0.502 | -0.370 | 0.714 | 0.229 | 2.746 |
|  |  |  |  |  |  |  |
| full time job (full time) | |  |  |  |  |  |
| full time non-geographic | 1.946 | 1.225 | 1.060 | 0.290 | 0.567 | 6.681 |
|  |  |  |  |  |  |  |
| Department (clinical) | |  |  |  |  |  |
| Non-clinical | 0.942 | 0.427 | -0.130 | 0.895 | 0.387 | 2.292 |
|  |  |  |  |  |  |  |
| Enough time for family (never or rarely) | | |  |  |  |  |
| quite often or very often | 0.447 | 0.165 | -2.180 | 0.029 | 0.217 | 0.922 |
|  |  |  |  |  |  |  |
| Intent of turnover (no) | |  |  |  |  |  |
| yes | 6.229 | 2.785 | 4.090 | 0.000 | 2.594 | 14.961 |
|  |  |  |  |  |  |  |
| Ward change (no) | |  |  |  |  |  |
| yes | 1.665 | 0.741 | 1.150 | 0.252 | 0.696 | 3.983 |
|  |  |  |  |  |  |  |
| Intent of migration (no) | |  |  |  |  |  |
| yes | 2.280 | 0.893 | 2.100 | 0.035 | 1.058 | 4.912 |
|  |  |  |  |  |  |  |
| Job offer to offspring(no) |  |  |  |  |  |  |
| yes | 0.721 | 0.263 | -0.900 | 0.371 | 0.352 | 1.475 |
|  |  |  |  |  |  |  |
| Health problems (no) | |  |  |  |  |  |
| yes | 3.117 | 1.341 | 2.640 | 0.008 | 1.341 | 7.244 |
|  |  |  |  |  |  |  |
| Suicide (no) |  |  |  |  |  |  |
| yes | 2.617 | 3.403 | 0.740 | 0.459 | 0.205 | 33.465 |
|  |  |  |  |  |  |  |
| Violence (no) | |  |  |  |  |  |
| yes | 2.629 | 1.047 | 2.430 | 0.015 | 1.205 | 5.737 |
|  |  |  |  |  |  |  |
| Number of death patients (3 or less) | |  |  |  |  |  |
| between 4 to 10 | 2.634 | 2.178 | 1.170 | 0.242 | 0.521 | 13.319 |
| 11 or more | 2.953 | 2.311 | 1.380 | 0.166 | 0.637 | 13.690 |
|  |  |  |  |  |  |  |
| constant | 0.278 | 0.509 | -0.700 | 0.485 | 0.008 | 10.038 |

**Table S3. RMSE for the neural network.**

|  | Output: Burnout | | Output: Low well-being | |
| --- | --- | --- | --- | --- |
| Network | Training | Testing | Training | Testing |
|  | 0.392 | 0.342 | 0.448 | 0.417 |
|  | 0.348 | 0.347 | 0.368 | 0.427 |
|  | 0.448 | 0.337 | 0.483 | 0.412 |
|  | 0.432 | 0.342 | 0.413 | 0.420 |
|  | 0.283 | 0.353 | 0.389 | 0.423 |
|  | 0.417 | 0.338 | 0.400 | 0.421 |
|  | 0.381 | 0.343 | 0.468 | 0.416 |
|  | 0.268 | 0.354 | 0.448 | 0.416 |
|  | 0.339 | 0.347 | 0.527 | 0.407 |
|  | 0.379 | 0.343 | 0.493 | 0.411 |
| Mean | 0.368 | 0.345 | 0.444 | 0.417 |
| Std. Deviation | 0.060 | 0.006 | 0.050 | 0.006 |
